# Revisiting the “obsessional slowness” syndrome

**DOI:** 10.1192/j.eurpsy.2021.1964

**Published:** 2021-08-13

**Authors:** C. Pedro Fernandes, M. Mangas, B. Jorge, D. Freitas

**Affiliations:** 1 Psychiatry, Hospital de Braga, Braga, Portugal; 2 Serviço De Psiquiatria, Unidade de Saúde Local do Baixo Alentejo, Beja, Portugal; 3 Serviço De Psiquiatria, Hospital de Braga, Braga, Portugal

**Keywords:** obsessional slowness, obsessive compulsive disorder

## Abstract

**Introduction:**

Obsessional slowness (OS) is a rare condition of disabling slow motor performance, first described in 1974, by Rachman, who documented 10 cases of “primary obsessional slowness”. Rachman argued that, although his patients with OS had Obsessive Compulsive Disorder (OCD), their motor symptoms were not related to the presence of motor-slowness-triggering obsessions/compulsions (e.g. checking and mental rituals). Whether OS truly is a distinct and “primary” entity is still a controversial issue, however.

**Objectives:**

To present and discuss the phenomenology of OS.

**Methods:**

Case reports of OS published in the literature, including Rachman’s descriptions.

**Results:**

The literature on OS is extremely limited, with no published, large-scale descriptive studies or randomized controlled trials. Some authors doubt that OS is a “primary” condition, pointing out the clear overlap between OS and catatonia and emphasizing that the latter disorder also occurs in non-schizophrenic patients, for example, ones with OCD. Additionally, OCD and depression often co-occur. Thus, in severe cases, it may be challenging to disentangle the separate contribution of both disorders to psychomotor slowness. It is also crucial to exclude the possibility that a patience has juvenile parkinsonism or other causes of motor slowness before diagnosing him/her with OS, given that the diagnostic approaches and treatment strategies for OS and the aforementioned disorders differ.

**Conclusions:**

OS seems to be a rare but often disabling motor manifestation of OCD, rather than a primary disease entity. However, some cases sit on the edge of current diagnostic criteria. Future research should help define OS more precisely.

**Disclosure:**

No significant relationships.

